# Oral administration of human carbonic anhydrase I suppresses colitis in a murine inflammatory bowel disease model

**DOI:** 10.1038/s41598-022-22455-y

**Published:** 2022-10-26

**Authors:** Kazuhiro Tange, Sen Yagi, Eiji Takeshita, Masanori Abe, Yasunori Yamamoto, Hideomi Tomida, Tomoe Kawamura, Masakazu Hanayama, Bunzo Matsuura, Yoshiou Ikeda, Yoichi Hiasa

**Affiliations:** 1 Department of Gastroenterology and Metabology, Ehime University Graduate School of Medicine, 454 Shitsukawa, Toon-Shi, Ehime, 791-0295 Japan; 2 Department of Internal Medicine, Saiseikai Matsuyama Hospital, Ehime, Japan; 3 Department of Inflammatory Bowel Diseases and Therapeutics, Ehime University Graduate School of Medicine, Ehime, Japan; 4 Department of Lifestyle-Related Medicine and Endocrinology, Ehime University Graduate School of Medicine, Ehime, Japan

**Keywords:** Gastrointestinal diseases, Inflammatory bowel disease

## Abstract

The incidence of inflammatory bowel disease (IBD) is increasing; hence, effective treatments are warranted. The therapeutic effect of human carbonic anhydrase I (hCA I) in IBD remains unknown. Therefore, we investigated whether oral tolerization to hCA I would induce antigen-specific protection from intestinal inflammation in vivo. Severe combined immunodeficient mice received hCA I, keyhole limpet hemocyanin (KLH), or phosphate-buffered saline (PBS) orally for 7 days. Colons and mesenteric lymph nodes (MLNs) were collected 4 weeks after cell transfer. Additionally, the mechanisms underlying the therapeutic effects were investigated. The comparison between the effects of well-established drugs and hCA I oral administration was investigated. Oral administration of hCA I ameliorated colitis remarkably. hCA I reached the cecum and ameliorated colitis more effectively than mesalazine and similarly to prednisolone. Compared with PBS treatment, hCA I treatment reduced interleukin (IL)-17a, IL-6, and retinoic acid-related orphan receptor gamma t (RORγt) expression in the colon or MLNs; moreover, hCA I markedly reduced IL-6, IL-17, and interferon-gamma (IFN-γ) levels in the MLN. Oral administration of hCA I induced immune tolerance and suppressed colitis in vivo. Thus, hCA I administration could be proposed as a new treatment option for IBD.

## Introduction

Inflammatory bowel diseases (IBD), including ulcerative colitis (UC) and Crohn's disease (CD), are chronic inflammatory disorders of the gastrointestinal tract commonly occurring in genetically susceptible individuals exposed to some risk factors^1^. Cases of these diseases, which develop in combination with environmental factors, are increasing annually and are becoming a social challenge^2,3^. However, the pathogenesis of IBD is not fully understood.

In recent years, both biologic therapies (e.g., anti-TNF-α, JAK inhibitor, α4β7 integrin inhibitor, and the interleukin (IL)-12 and IL-23 p40 subunit inhibitor) and the disease control rate have been overall improved. However, existing drugs still cause several side effects^4,5^. It has been reported that the target approach for IBD, especially for CD and UC, is important in improving long-term prognosis. Additionally, this approach is necessary to better understand the condition and allow for treatment based on the individual and time of onset^6,7^. From this perspective, a critical need for treatment options with few side effects prevails.

Uncontrolled innate and adaptive immune responses to symbiotic bacteria, self-antigens, and various microbial products and foodstuffs are etiological factors of IBD^8^. Previous studies have evidenced that the fecal extract cecal bacterial antigen (CBA) is associated with its etiology^9,10^. We have previously identified that carbonic anhydrase I (CA I; 261 amino acids, AA; 29 kilodaltons) is a major protein of CBA and an antigen involved in the induction of the immune response^11^. Within the gastrointestinal tract, CA I is expressed only in the colon and plays a central role in acid–base balance, pH regulation, and CO_2_ transport^12^. In addition, the expression of CA I was reduced in the active stage of experimental colitis in mouse models and patients with UC, whereas its expression increased in the remission stage^13^. Therefore, it was considered that the decrease in CA I expression might be correlated with disease activity. We previously reported that the oral administration of mouse CA I (mCA I) improves IBD in murine models via antigen-specific immune tolerance^14^. Moreover, we provided evidence that the mCA I epitope can drive immune tolerance and demonstrated that regulatory dendritic cells pulsed with the mCA I peptide (DCregsCA I peptide) induce antigen-specific protection against colitis in murine models of IBD^15^. Oral tolerance has been reported in various diseases^16^. However, as humans and mice possess different AA sequences, it is essential to confirm the therapeutic effect of humanized proteins for clinical application. In this study, we investigated oral immunization using human CA I (hCA I) as an immunotherapy method and evaluated its efficacy in a murine colitis model. Furthermore, we compared the therapeutic effects of hCA I with those of established drugs.

## Results

### Oral sensitization using hCA I ameliorates experimental colitis in the mouse model

**Figure 1:**
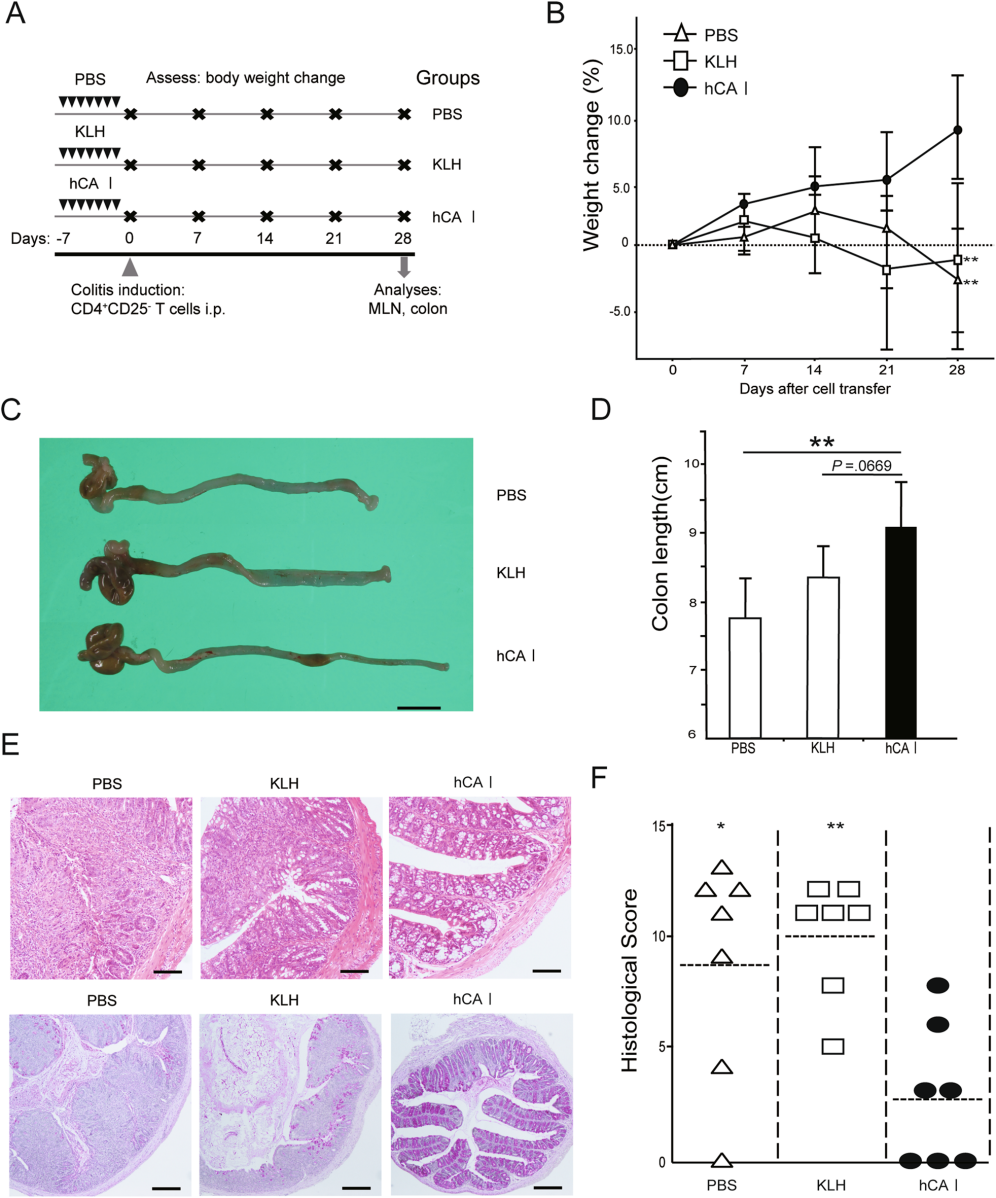
Oral tolerization using hCA I protects mice against experimental colitis. (**A**) Experimental protocol (see “Methods”). (**B**) Relative changes in body weight (%) over time at 0, 7, 14, 21, and 28 days. CD4^+^CD25^−^ T cell (3 × 10^5^) transfer model mice (n = 7 mice/group) were treated with PBS, KLH, and hCA I indicated as triangles, squares, and black circles, respectively. The error bars indicate SD. (**C**) Macroscopic findings of the colon on day 28. Scale: 10 mm. (**D**) Colon lengths in colitic mice on day 28 (n = 7). (**E**) Histological analysis of the colon on day 28. PBS- and KLH-treated mice demonstrated inflammatory cell infiltration, marked increase in mucosal height, and severe loss of goblet cells. Oral administration of hCA I improved these findings. Representative images at high (H&E stain; original scale: 100 μm) and low (periodic acid Schiff staining; original scale: 200 μm) magnifications are shown. (**F**) Histological scores on day 28. Horizontal bars: median. *P < 0.05; **P < 0.01 (vs. hCA I-treated mice). Data shown are representative of two independent experiments.

We have previously provided evidence that oral administration of the mCA I antigen has a suppressive effect on enteritis in Dextran sulfate sodium (DSS) model and CD4^+^CD25^−^ T cell transfer model mice^14^. To assess the clinical application of orally administered CA I for immunotherapy, hCA I was prepared, and further experiments were conducted (Supplementary Fig. S1). We analyzed whether oral sensitization using hCA I ameliorates inflammatory levels in the CD4^+^CD25^−^ T cell transfer model of colitis. Mice were administered hCA I as described in the “Methods” (Fig. 1A). KLH is a protein that has long attracted attention as an inducer of oral immune tolerance^17^; however, it has not been clinically effective in IBD patients (CD and UC)^18^. Therefore, KLH was used as a control protein for the purpose of assessing immune tolerance. The usual diet of the mice consisted of a normal diet containing the protein. We assessed body weight change and demonstrated that the hCA I-treated group experienced minimal weight loss 4 weeks after colitis was induced compared with that of the PBS- and KLH-treated groups (*P* < 0.01; Fig. 1B). Four weeks after cell transfer, colon length was significantly greater in mice treated with hCA I than in PBS-treated mice (*P* < 0.01; Fig. 1C and D). Histological examination revealed that colitis was characterized by massive infiltration of inflammatory cells, elongation and degeneration of the crypt, epithelial hyperplasia, and depletion of goblet cells, all of which were suppressed in the hCA I group, even 4 weeks post cell transfer. KLH- and PBS-treated mice injected with CD4^+^CD25^−^ T cells experienced severe colitis. Histological scores were significantly lower in mice treated with hCA I than in KLH- or PBS-treated mice (*P* < 0.05; Fig. 1E,F).

### Oral administration of hCA I reduces inflammatory cytokine response

**Figure 2:**
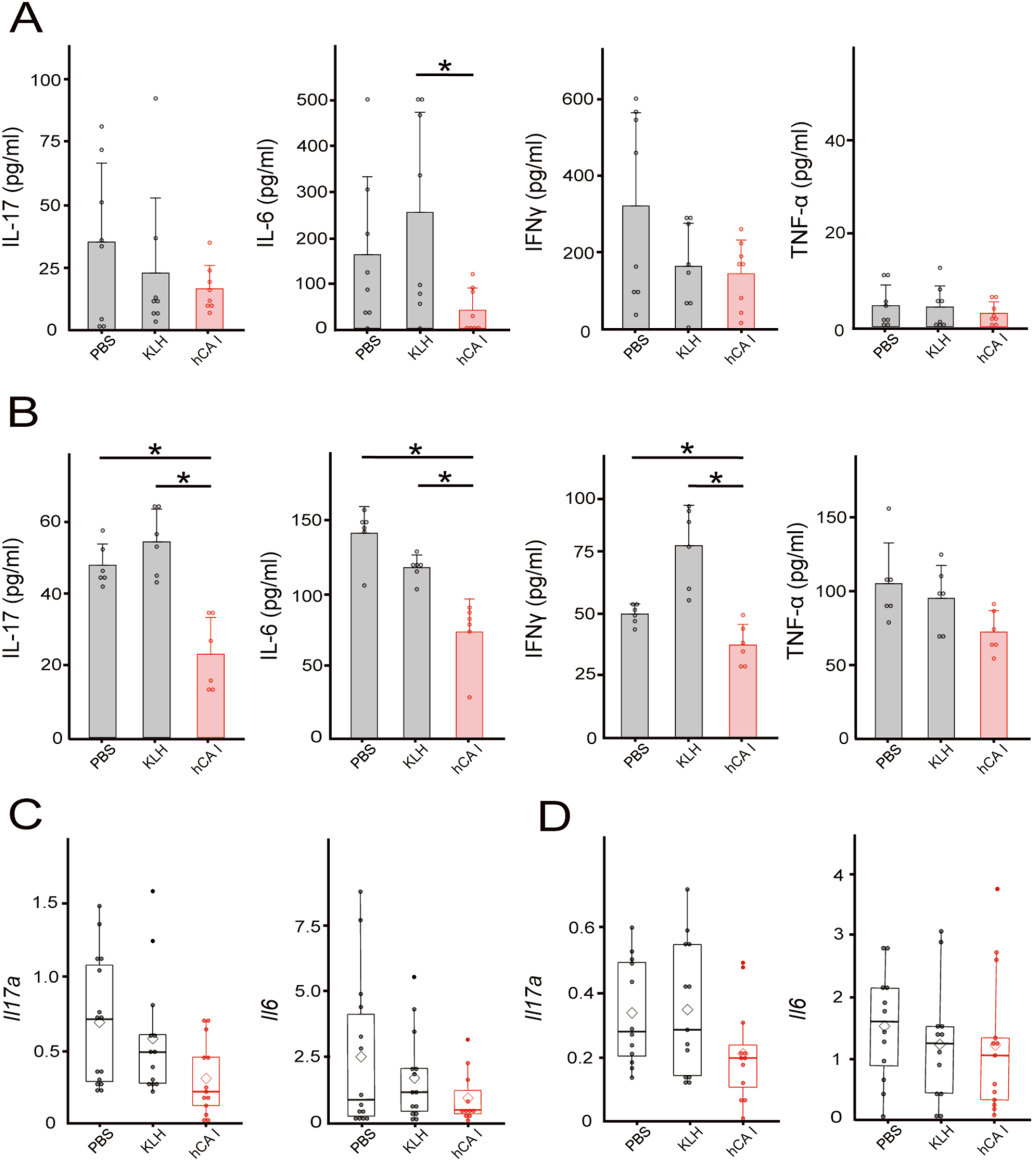
Treatment with orally administered hCA I suppressed inflammatory responses in the colon and MLNs in experimental colitis model mice. Four weeks after CD4^+^CD25^−^ T cells and DCs were transferred, the colons and MLNs were collected. (**A**) the colons, cultured ex vivo for 72 h, were measured using ELISA; mean ± SD of 8 mice/group. (**B**) Secreted cytokine concentrations from MLN cells (1 × 10^6^), cultured with PMA and ionomycin for 72 h, were measured using ELISA; mean ± SD of 6 mice/group; *P < 0.05. (**C**) Four weeks after CD4^+^CD25^−^ T cells were transferred, transcription factor or cytokine mRNA expression levels in the colon were quantified using real-time RT-PCR; mean ± SD of 14–15 mice/group. (**D**) Transcription factor or cytokine mRNA expression levels in MLN cells were measured using real-time RT-PCR; white diamond indicates the mean, horizontal lines indicate the median, line indicate ± SD of 12–13 mice/group. *P < 0.05.

We have previously clarified that the oral administration of mCA I induced antigen-specific protection of inflammatory cytokines, such as IL-17, IL-6, and TNF-α^14^. Therefore, we decided to investigate whether administration of the humanized protein would demonstrate a similar reaction. The expression of cytokines in the colon and MLNs was measured to investigate the mechanisms by which the oral administration of hCA I ameliorates colitis in the T cell transfer model. Production of IL-6 was significantly lower in the colon (Fig. 2A), and IL-6, IL-17 and IFN-γ production was significantly lower in the MLNs (*P* < 0.05) of mice administered hCA I (Fig. 2B). Thereafter, transcripts were also evaluated. In the colon of hCA I-treated mice, the expression of IL-17A showed a reduction trend (*P* = 0.0528) compared with that in PBS-treated mice (Fig. 2C). There was a similar trend, though not significant, at the MLNs level (Fig. 2D).

### Orally administered hCA I reaches the large intestine

**Figure 3:**
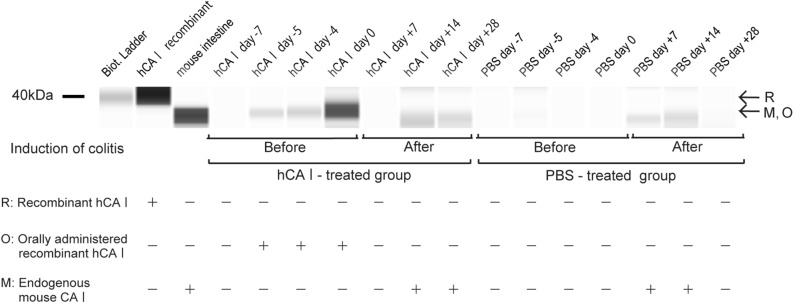
Quantification of hCA I or mouse CA I in feces. Changes in CA I protein (including mouse CA I and recombinant hCA I) content in feces before and after oral administration (PBS: control group) detected using immunoblot. *R* recombinant hCA I; *O* orally administered hCA I; *M* mouse CA I.

To clarify how the orally administered hCA I protein behaves in vivo, first, stools were collected daily from three mice from the hCA I and PBS groups. Thereafter, endogenous mCA I, which was shed due to colitis, or orally administered hCA I proteins were collected from the feces of mice using a polyclonal antibody. Finally, the amount of CA I contained in the fecal samples obtained from each group was measured using immunoblotting (see “Methods”). Fecal samples collected from the orally administered hCA I group before the induction of colitis demonstrated a shortened protein band, cleaved by the enterokinase recognition unit, in comparison with that of recombinant hCA I (Fig. 3; R and O). Upon producing a recombinant protein containing an enterokinase recognition sequence, we were able to demonstrate that CA I contained in feces was not contaminated by spillage caused by mice (Supplementary Fig. S1). In contrast, in the negative control group (before oral administration of PBS), the protein band was negative. After the induction of colitis, a band of mCA I protein, which was exfoliated from the epithelial cells of the large intestine, appeared (Fig. 3; M). On day 7, mCA I was not detected in the feces of mice from the hCA I-administered group, whereas this band had already appeared in the PBS group. In addition, on day 28, the chain disappeared in the PBS-treated group but continued to appear in the feces of hCA I-treated mice. This result suggested that colitis developed earlier in the PBS group. Moreover, the amount of CA I in the intestinal tract, as well as the feces, decreased in the PBS group on day 28 when colitis was in progression. Upon analyzing the immunoblot results, we found many nonspecific bands and smears in both hCA I and PBS-treated mice (Supplementary Fig. S2A). To determine whether these bands are representative of the fecal hCA I concentrations of this study, we performed similar experiments with isotype controls and those immunoprecipitated with other CA I antibodies and confirmed that the detected CA I band is specific (Supplementary Fig. S2B,C).

### Rectal sensitization with hCA I suppressed colonic inflammation in a mouse model of experimental colitis during the chronic inflammation phase

**Figure 4:**
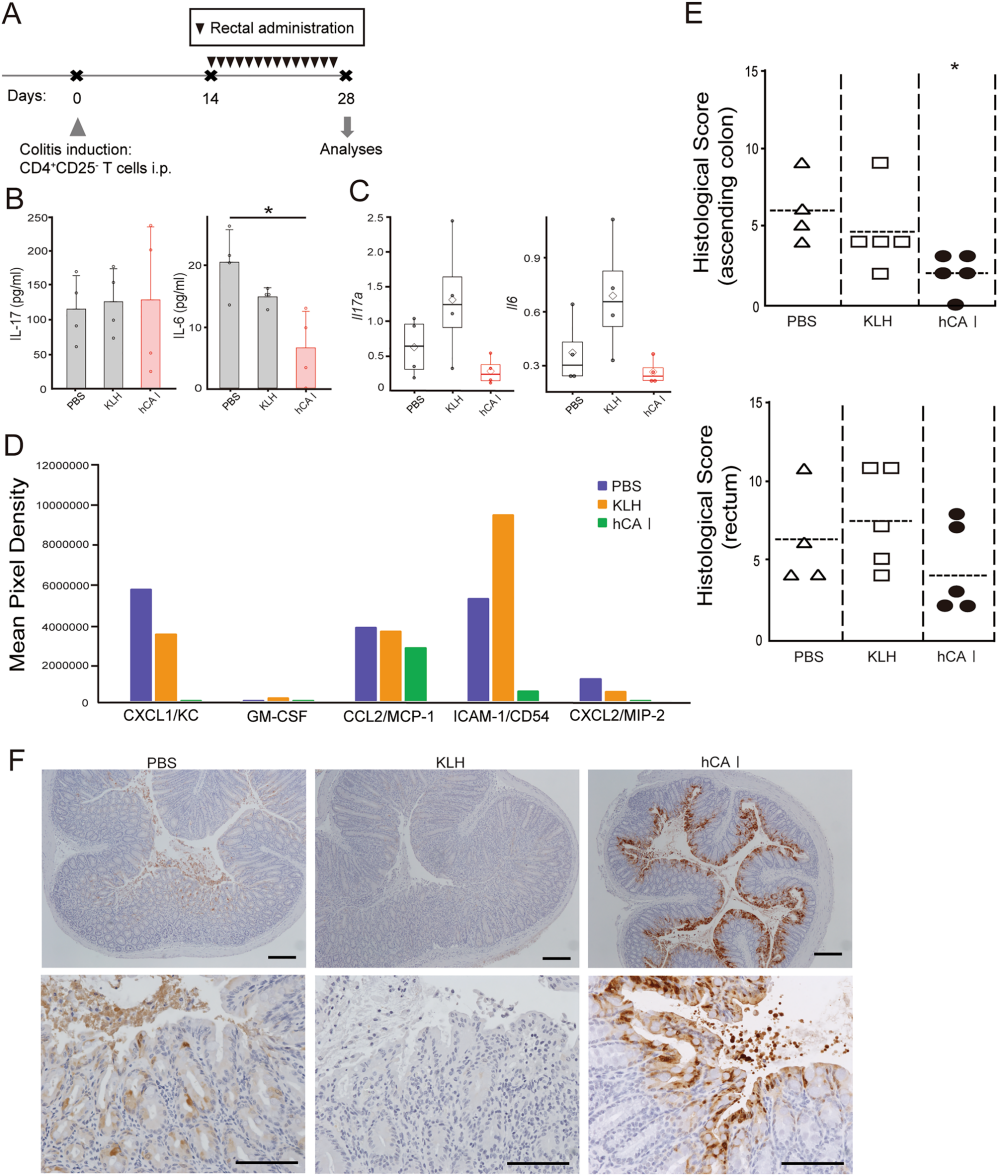
Rectal administration of hCA I after induction of colitis resulted in the suppression of colitis development. (**A**) Experimental protocol (see “Methods”). Four weeks after CD4^+^CD25^−^ T cells were transferred, the MLNs were collected. (**B**) Secreted cytokine concentrations from MLN cells (1 × 10^6^), cultured with PMA and ionomycin for 72 h, were measured using ELISA; mean ± SD of 4 mice/group. *P < 0.05. (**C**) Transcription factor or cytokine mRNA expression levels in MLN cells were measured using real-time RT-PCR; white diamond indicates the mean, horizontal lines indicate the median, line indicate ± SD of 4 mice/group. *P < 0.05. (**D**) Secreted cytokine concentrations from colons were measured using Mouse Cytokine Array; the supernatants (n = 3) collected from each group were mixed. (**E**) Histological scores on day 28. Horizontal bars: median; *P < 0.05 (vs. PBS-treated mice). Left; ascending colon, Right; rectum. CD4^+^CD25^−^ T cell (3 × 10^5^) transfer model mice (n = 4–5 mice/group) were treated with PBS, KLH, and hCA I indicated as triangles, squares, and black circles, respectively. One mouse in the PBS group died due to the procedure during intraperitoneal administration of CD4^+^CD25^−^ T cells. (**F**) CA I expression was evaluated using immunohistochemical staining. Representative images at high (original scale, 200 μm) and low (original scale, 100 μm) magnifications are shown. Data shown are from single experiments.

As the large intestine is covered with epithelium and mucus, we conducted rectal administration experiments to clarify whether orally administered hCA I acts in the small intestine or the large intestine. We identified that CA I in feces decreased during the colonic inflammation progression; hence, we decided to further investigate whether sensitization with hCA I from the rectum during CA I decrease inhibits colonic inflammation. When 200 μL of an Indigocarmine-stained drug was administered to mice using a rectal administration tube, it was confirmed that the drug reached the ascending colon (Supplementary Fig. S3A). As per the oral administration, hCA I, KLH, and PBS were administered daily for 7 days before the induction of colitis, where no suppression of colitis was observed (Supplementary Fig. S3B,C). Next, we conducted an experiment in which hCA I was administered rectally on days 14–27 when CA I in feces decreased due to colonic inflammation (Fig. 4A). In the MLNs, we observed a decrease in pro-inflammatory cytokines production, with a considerable difference for IL-6 compared with that in PBS-treated mice (Fig. 4B). Consequently, transcripts were evaluated. In the MLNs of hCA I-treated mice, the expression of IL-17A and IL-6 showed reduction trends (*P* = 0.0741 and 0.0578, respectively) compared with those of KLH-treated mice (Fig. 4C). In addition, a cytokine array was used to investigate whether rectally administrated hCA I has acted on any other important targets to influence the intestinal tract. Cytokines and chemokines involved in innate and adaptive immunity of the colon tended to be expressed lower in the hCA I group than in the other groups (Fig. 4D and Supplementary Fig. S4). Histological scores in the ascending portion of the colon were significantly lower in mice treated with hCA I than in those treated with PBS (*P* < 0.05; Fig. 4E). Immunostaining of the ascending portion of the colon tissues showed that epithelial CA I was preserved in the hCA I-treated group (Fig. 4F).

### Oral sensitization using hCA I demonstrated a non-inferiority suppressive effect in the experimental colitis mouse model compared with established drugs for IBD

**Figure 5:**
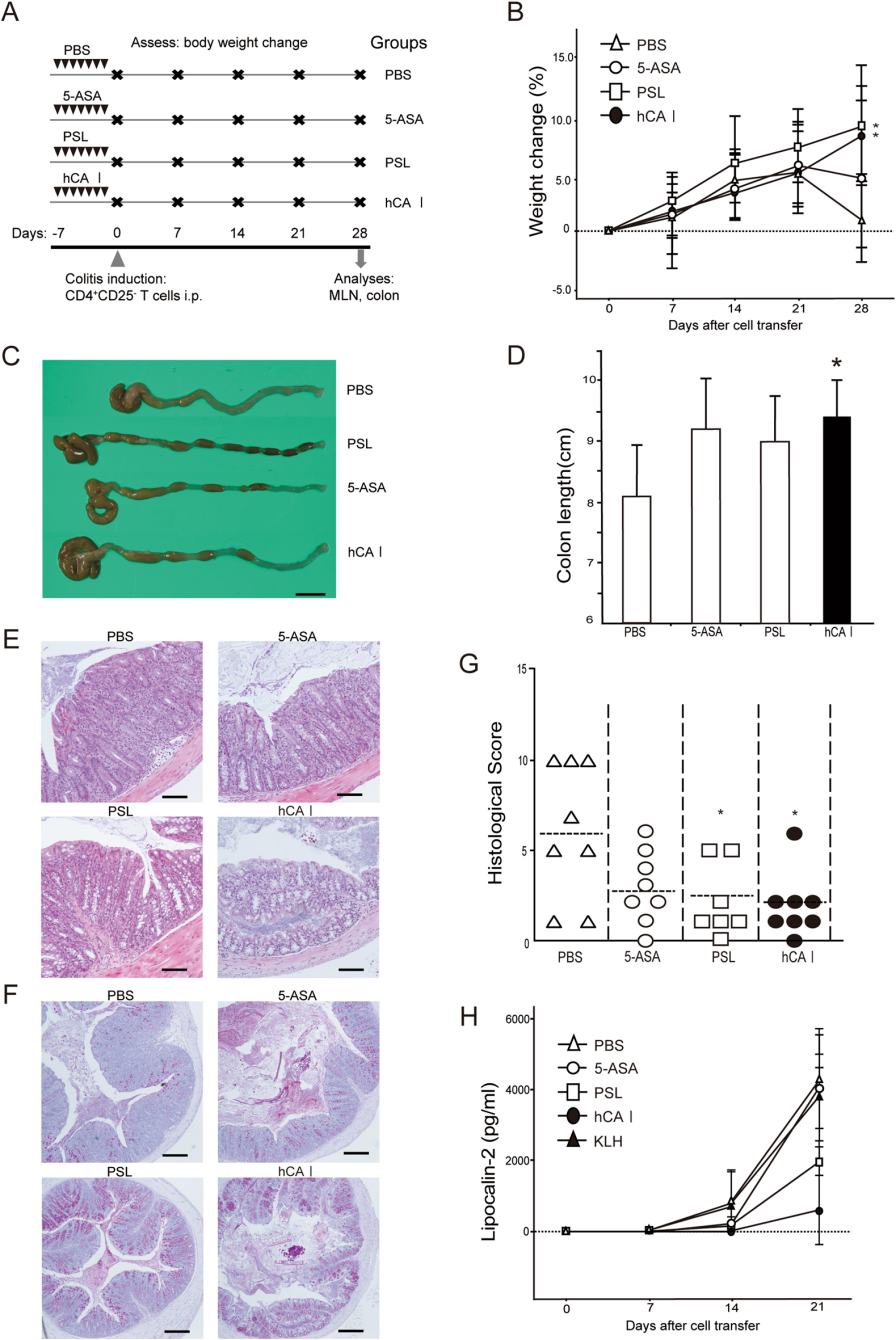
Experimental comparison of therapeutic effects of hCA I with pilot drugs. (**A**) Experimental protocol. Groups: PBS, mesalazine (5-ASA) 100 mg/kg per os (p.o.), prednisolone (PSL) 2 mg/kg p.o., and human CA I (hCA I) 0.3 mg/d p.o. (see “Methods”). (**B**) Relative changes in body weight (%) over time at 0, 7, 14, 21, and 28 days. CD4^+^CD25^−^ T cell (3 × 10^5^) transfer model mice (n = 8 mice/group) were treated with PBS, 5-ASA (mesalazine), PSL (prednisolone), or hCA I indicated by triangles, white circles, squares, and black circles, respectively. The error bars indicate SD. (**C**) Macroscopic findings of the colon on day 28. Scale: 10 mm. (**D**) Colon lengths in colitic mice on day 28 (n = 8). (**E**,**F**) Histological analysis of the colon on day 28. PBS- and 5-ASA-treated mice evidenced inflammatory cell infiltration, marked increase in mucosal height (H&E stain; original scale: 100 μm), and severe loss of goblet cells (periodic acid Schiff staining; original scale: 200 μm) magnifications are shown. Oral administration of hCA I and PSL improved these findings. (**G**) Histological scores on day 28. Horizontal bars: median; *P < 0.05 (vs. PBS-treated mice). Data shown are representative of two independent experiments. (**H**) Analysis of the fecal biomarker lipocalin-2. CD4^+^CD25^−^ T cell (3 × 10^5^) transfer model mice (n = 3 mice/group) were treated with PBS, 5-ASA (mesalazine), PSL (prednisolone), hCA I, or KLH indicated by triangles, white circles, squares, black circles, and black triangles, respectively.

Although CD4^+^CD25^−^ T cell transfer model mice are frequently used as a preclinical model for IBD, our study has an added advantage as we used this chronic colitis model to compare the efficacy of established drug treatments, namely 5-ASA and PSL, with that of hCA I. Mice were administered hCA I or established drugs (5-ASA and PSL) and colitis was induced (Fig. 5A). We assessed body weight change and identified that the hCA I- and PSL-treated groups experienced minimal weight loss 4 weeks after colitis was induced compared with that of the PBS group (*P* < 0.05; Fig. 5B). Four weeks after cell transfer, colon length was significantly greater in mice treated with hCA I than in PBS-treated mice (*P* < 0.05; Fig. 5C,D). Histological examination revealed that colitis was suppressed in the hCA I and PSL groups 4 weeks after cell transfer, but PBS-treated mice experienced severe colitis. Additionally, histological scores were significantly lower in mice treated with hCA I or PSL compared with those in PBS-treated mice (*P* < 0.05; Fig. 5E–G). Analysis of the fecal biomarker lipocalin 2 over time showed a suppression trend of colitis in hCA I over the other groups (Fig. 5H).

### Oral tolerization using hCA I showed a non-inferior suppressive effect on inflammatory cytokines compared with established drugs

**Figure 6:**
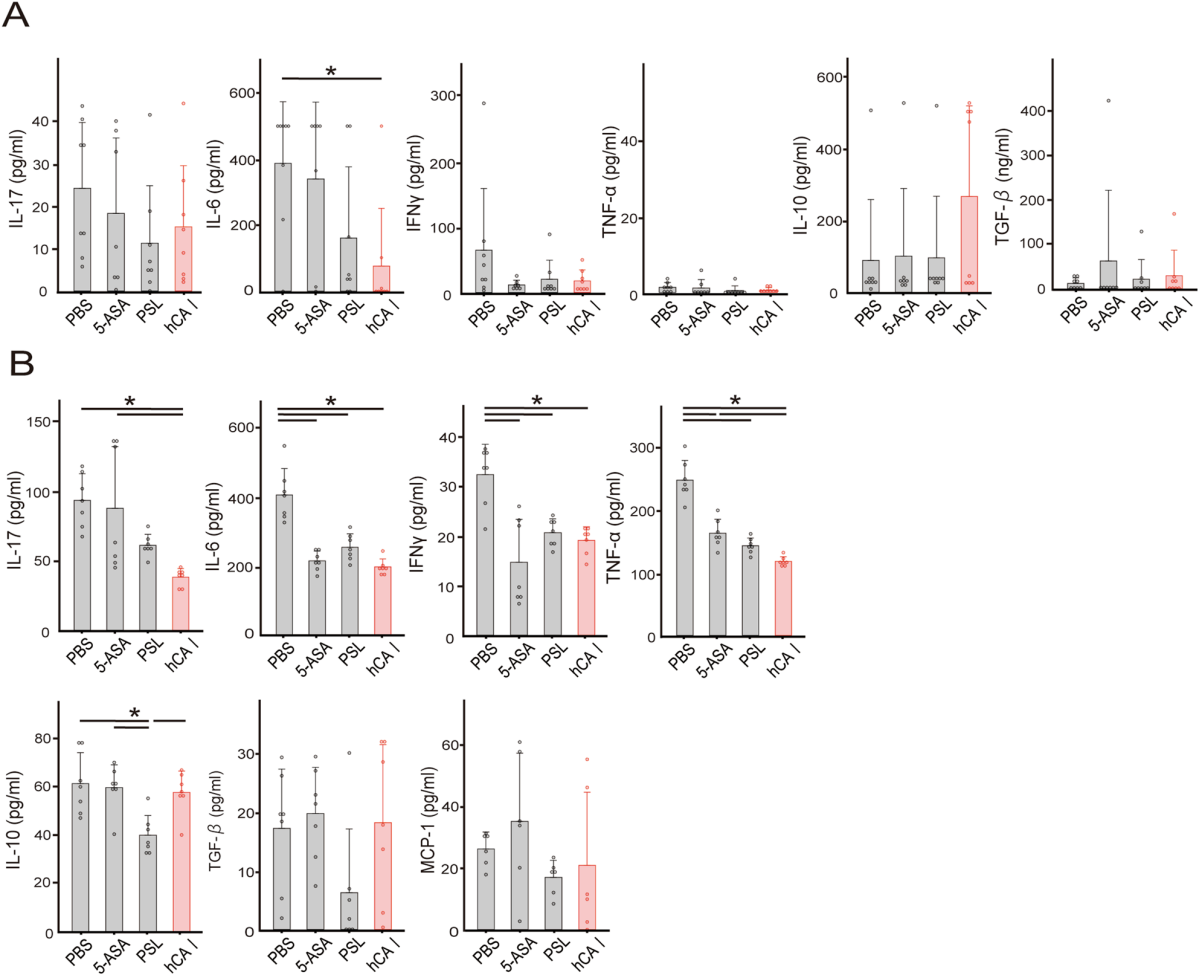
Inflammatory responses in the colon and MLNs in experimental colitis model mice. Four weeks after CD4^+^CD25^−^ T cells and DCs were transferred, the colon and MLNs were collected. (**A**) Secreted cytokine concentrations from the colon, cultured ex vivo for 72 h, were measured using ELISA; mean ± SD of 7–8 mice/group. (**B**) Secreted cytokine concentrations from MLN cells (1 × 10^6^), cultured with PMA and ionomycin for 72 h, were measured using ELISA; mean ± SD of 6 mice/group. *P < 0.05.

**Figure 7:**
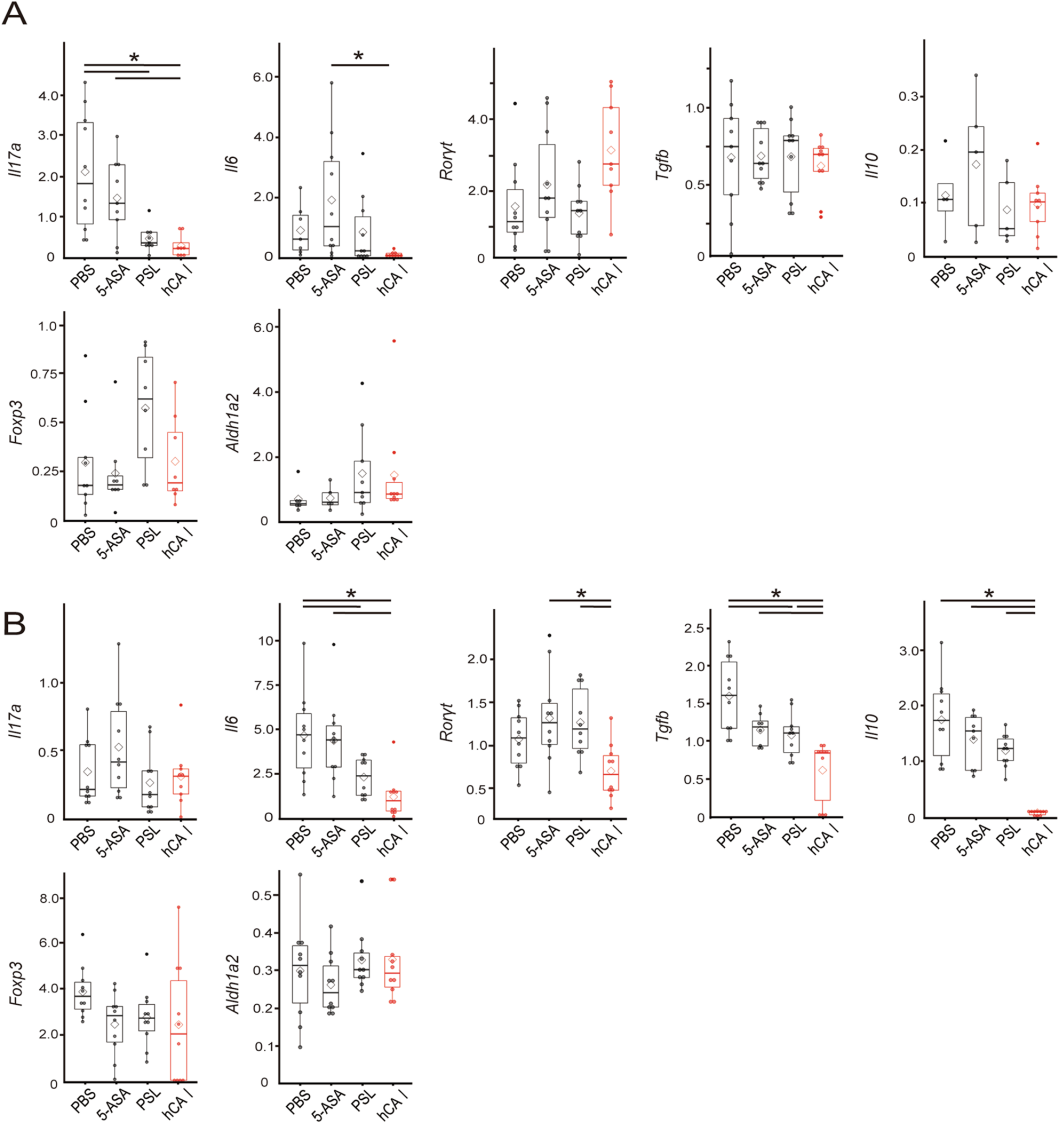
Treatment with orally administered hCA I suppressed inflammatory responses in the colon and MLNs of colitic mice. (**A**) Four weeks after CD4^+^CD25^−^ T cells were transferred, transcription factor or cytokine mRNA expression levels in the colon were quantified using real-time RT-PCR; white diamond indicates the mean, horizontal lines indicate the median, line indicate ± SD of 6–11 mice/group. (**B**) Transcription factor or cytokine mRNA expression levels in MLN cells were measured using real-time RT-PCR; mean ± SD of 8–10 mice/group. *P < 0.05.

We previously clarified that oral sensitization using mouse CA I induces CD103^+^CD11c^+^ DCs and forkhead box protein 3 (Foxp3)^+^CD4^+^CD25^+^ T cells and suppresses Th17 cell responses in the MLNs^14^. Furthermore, we identified that the mCA I 58–73 epitope can interact with major histocompatibility complex (MHC) class II molecules and evidenced DCregsCA I peptide-induced antigen-specific protection against colitis via a similar mechanism in a murine IBD model^15^. In this study, we investigated the effects of the oral administration of hCA I on transcripts and cytokines involved in colitis. Production of IL-6 was significantly lower in the colon of hCA I-administered mice, as opposed to the 5-ASA and PSL groups (*P* < 0.05) (Fig. 6A). In the MLNs, hCA I suppressed the production of inflammatory cytokines, including IL-6, IL-17, IFN-γ, and TNF-α, more effectively than 5-ASA (*P* < 0.05; Fig. 6B). Thereafter, we assessed the expression of transcripts, such as ALDH1A2 (involved in Treg induction via CD103^+^ DCs), TGF-β, and IL-10 in the MLNs and evaluated their involvement in the induction of oral tolerance. The expression of IL-17A was significantly reduced (*P* < 0.05), whereas ALDH1A2 and Foxp3 tended to increase in the colons of hCA I-treated mice compared with those in the colons of PBS-treated mice. Additionally, the expression of IL-6 was significantly reduced in the colons of hCA I-treated mice compared with that in 5-ASA-treated mice (*P* < 0.05; Fig. 7A). Moreover, IL-6 expression levels were significantly lower in the MLNs of mice treated with hCA I and PSL compared with those in PBS-treated mice (*P* < 0.05). In addition, IL-6 and retinoic acid-related orphan receptor gamma t (RORγT) expression levels were significantly reduced in the MLNs of hCA I-treated mice compared with those in 5-ASA-treated mice (*P* < 0.05). The expression levels of ALDH1A2 in the MLNs were not statistically significant but were higher in hCA I-treated mice compared with the other groups (Fig. 7B). On the other hand, the induction of Tregs due to oral administration of mCA I, as shown in previous studies^14^, was not observed in the MLNs and spleen on day 28 using flow cytometry (Supplementary Fig. S5). In order to clarify the relationship between hCA I and intestinal bacteria, we examined the changes in intestinal bacteria in feces on day 0. In the hCA I groups, an increase in the percentage of *Bacteroidetes* at the phylum was observed (Supplementary Fig. S6A). As per the alpha diversity analysis, the PBS group tended to have lower diversity, with no marked change in each group (Supplementary Fig. S6B). In principal component analysis with β diversity, all groups showed different characters (Supplementary Fig. S6C).

## Discussion

We have previously identified that the major cecal bacterial antigen, CA I, is a target antigen for IBD^11^. Furthermore, immunotherapy using DCregs and oral administration of mCA I improve antigen-specific immune tolerance in murine IBD models^11,14^. Moreover, we identified the mCA I epitope and demonstrated that DCregsCA I peptide induced antigen-specific protection against colitis in a murine model of IBD^15^. The three main findings of this study are as follows: (1) hCA I-specific oral tolerance was inducible in a murine model of IBD and inhibited the progression of murine experimental colitis by suppressing inflammatory cytokines in the MLNs; (2) Upon colitis worsening, the amount of fecal CA I decreases, and rectal supplementation with hCA I can reduce the severity of colitis; (3) hCA I had a non-inferior suppressive effect on colitis compared with established drugs (5-ASA or PSL) in a murine model of IBD.

KLH is a protein that has long been of interest in mouse models as an oral immune tolerance inducer. However, KLH has had poor clinical efficacy in IBD patients (CD and UC)^17,18^. Margalit et al.^19^ attributed this discrepancy to the short duration of KLH administration, the possible influence of other antigens, and the fact that KLH was used as a parameter for immune tolerance and T cell proliferation, but it is unclear which T cells and Tregs respond to KLH stimulation. We previously reported that oral administration of mCA I was upregulated in the differentiation of Foxp3 + Tregs from Foxp3-CD4 + CD25- T cells in the MLNs and colons of CA I-treated mice compared with KLH- or PBS-treated mice^14^. These results suggest that CA I-specific oral tolerance is induced in IBD and prevents progression of colitis by inducing antigen-specific Foxp3 + CD4 + CD25 + T cells. In this study, KLH was used as a control protein, consistent with our previous studies. hCA I has accumulated stronger evidence than KLH and demonstrated its efficacy in suppressing colitis for clinical application. Furthermore, we considered different proteins of the same mass as that of hCA I or scrambled sequences as controls. However, the possibility of antigenic active sites appearing when the protein was degraded to amino acids prevailed.

Oral administration of hCA I has the same suppressive effect as that due to the oral administration of mCA I in T cell transfer model mice (Fig. 1). Oral administration of hCA I reproducibly suppressed colitis and weight loss in vivo (Figs. 1 and 5), as well as the production of inflammatory cytokines and suppression of transcripts related to inflammation in vitro (Figs. 2, 6, and 7).

Previous reports show that regulatory T cells (Tregs) that differentiate outside the thymus maintain intestinal homeostasis by suppressing the function and proliferation of inflammatory cells via the anti-inflammatory cytokine IL-10^20,21^. Extrathymic differentiation of Tregs also influences the composition of the microbiota and suppresses Th2 inflammation, including allergic inflammation at the mucosal interface^22^. In addition, expanding DCs in vivo enhance the induction of oral tolerance^23^, whereas gut mucosal CD103^+^ DCs induce Foxp3^+^ regulatory T cells via a TGF-β and retinoic acid-dependent mechanism^24–26^. We have demonstrated that immunotherapy using DCregs and the oral administration of mCA I increased the production of IL-10 and expression of transcripts, such as FOXP3 and AlDH1A2, occurring mainly in the MLNs. We have also demonstrated, using flow cytometry, the ability of mCA I to induce antigen-specific Tregs and CD103^+^ CD11c^+^ DCs on day 7 post colitis induction in murine models of IBD (DSS and T cell transfer model mice)^11,14,15^. In this experiment, an increase in the expression of ALDH1A2 involved in Treg induction via dendritic cells was confirmed (Fig. 7A,B), but no significant difference was observed upon mCA I administration^14^. Further, we measured the induction of Tregs on day 28 using flow cytometry and found no remarkable changes (Supplementary Fig. S5). Because of the difference in amino acid sequence between mouse and human CA I, the oral administration of hCA I may have weakened the effect of antigen-specific tolerance (Supplementary Table S1). However, inflammation of the colon was reproducibly suppressed (Figs. 1, 4, and 5). In fact, the inflammatory cytokines IL-6, IL-17, and IFN-γ were predominantly reduced in the MLNs of hCA I-treated mice compared with the control group (Figs. 2B and 6). In addition, transcripts of IL-6 and IL-17A underwent similar changes (Figs. 2C,D, and 7).

The IFN-γ-induced chemokines CXCL9 or CXCL10, and their common receptor CXCR3, are upregulated in the gut of IBD patients^27^, A recent study suggested that the myeloid-cell-specific adaptor protein, MyD88, signaling, as well as the cytokines IL-12, IL-23, and IFN-γ, are closely involved in the pathogenesis of colon inflammation in *NEMO*^*tamIEC-KO*^ mice^28^. In clinical practice, it has been proven that ustekinumab exerts a therapeutic effect in CD and UC, and the control of cytokines involved in Th1 responses, such as IL12 and IFN-γ, are increasing in significance^29^. Our data indicate that the production of IL-6, IL-17, and INF-γ were significantly lower in the MLNs of the hCA I-treated group (Figs. 2B and 6). Our previous studies reported similar results^11,14,15^. In this experiment, we mainly examined the cytokines involved in regulatory immunity, which we had identified in our previous experimental system for mCA I; however, it is necessary to further investigate the role of Th1 responses in T cell transfer model mice as it may be drastically involved in the development of colitis.

Orally administered proteins and amino acids regulate intestinal immunity. For instance, in mice fed a protein-deficient diet, the amount of Tregs in the small intestine are markedly reduced. This reaction is also observed in germ-free mice^30^. As demonstrated in sterile mice, tryptophan—an amino acid contained in food—was metabolized by intestinal bacteria to form kynurenine, which induces Treg activity^31^. Moreover, it has been reported that protein intake affects Treg differentiation in the small intestinal mucosa but not in the large intestinal mucosa^30^. On the contrary, most proteins in the diet exacerbated colitis in C56BL/6J specific pathogen-free mice with DSS-induced colitis^32^. Proteins that exhibit protective effects against colitis are rare. Previously, we determined that CB-17 SCID mice, injected with CD4^+^CD25^−^ cells containing DCregsCA I, experienced suppressed colitis, whereas those that received regulatory dendritic cells pulsed with cecal bacterial antigen depleted of CA I (DCregs_CBA-CA I_) experienced no colitis suppresion^11^. Therefore, CA I is a rare protein that induces immune tolerance in the large intestine. In this study, hCA I was measured via immunoblot and was more abundant in the feces than mouse CA I, which is shed from the colon due to colitis (Fig. 3 and Supplemental Fig. S2). Therefore, we believe a sufficient amount of hCA I protein was administered in this mouse model. A previous study revealed that DCs sample luminal contents by extending their protrusions through the epithelium without disrupting tight junctions^33^. From this perspective, orally administered hCA I could directly control the induction of immunity in the small intestinal mucosa or large intestine. However, direct administration of hCA I into the colon before induction of colitis did not suppress colonic inflammation. Our previous report demonstrated that oral administration of mCA I induced Tregs and DCregs in the MLNs^14^. In addition, we demonstrated that a T-cell epitope peptide derived from CA I could interact with MHC class II molecules; more specifically, mCA I 58–73 peptide-pulsed DCregs protected mice with experimental colitis^15^. These results suggest that the administered hCA I protein or partially degraded amino acid induced immunity in the small intestine. Furthermore, when comparing the amount of CA I protein in feces from PBS- and hCA I-treated groups, CA I was not found in feces of hCA I-treated mice, whereas it was already present on day 7 in PBS-treated mice. In addition, CA I amount decreased in the PBS group on day 28. These results show that colitis onset was suppressed in the hCA I-treated group; hence, the protein band was clearly observed, even on day 28 (Fig. 3). Although it has been reported that CA I expression was decreased in tissues of patients with UC and exacerbated colitis^13^, there have been no reports of a one-time increase in fecal CA I in the early stages of colitis or a depletion upon disease exacerbation. These results suggest the possibility that intestinal homeostasis cannot be maintained due to a lack of CA I, which induces immune tolerance during colitis exacerbation. In fact, supplementation with CA I during exacerbations of colonic inflammation suppressed colitis development (Fig. 4).

Upon comparing the effects of hCA I with established drugs, we revealed that hCA I ameliorated colitis more effectively than mesalazine and similarly to PSL (Fig. 5). Mucosal healing is critical in controlling IBD^34–36^. Various biologics have been developed, and improvements in IBD treatments have been made. However, mild and moderate cases are mainly treated using 5-ASA preparations, and few other treatment options are available. In addition, established drugs possess various side effects, such as secondary infections, and improved treatment options are, therefore, essential. The use of biologics can increase the risk of developing an infection, malignancy, immunological issues, and metabolic and hematologic complications^4^. Conversely, CA I is a self-antigen and is considered a therapeutic drug that is safe to use with a low possibility of causing allergic reactions. In addition, it has a completely different mechanism of action to that of existing drugs and could, therefore, augment therapeutic effects when used in combination.

As previously stated, through immunoprecipitation and immunoblotting, we identified a transient increase in fecal CA I levels in the early phase of colitis, followed by a decrease in fecal CA I levels in the exacerbation phase. We also observed that hCA I influenced the composition of intestinal bacteria (Supplemental Fig. S6). This suggests that CA I administration influences the intestinal microbiota content even during colitis. We hypothesized that similar changes in the intestinal microbiota composition occur transiently during colitis, although more studies, including analyses of intestinal bacteria and metabolites, are required to verify our hypothesis. In this study, we were able to clarify the role of CA I in the intestinal environment where various factors are intertwined. Moreover, CA I may also have the potential to serve as a biomarker to assess the activity of colitis. Despite this, we believe that additional experiments using KO mice colon-specific CA I are necessary to investigate the effects of host CA I. While there have been reports of CA I from colon-specific Cre-expressing mouse being used, there are currently no reports of CA I from KO mice.

## Conclusion

We identified that CA I, present only in the large intestine within the gastrointestinal tract^12^, is an antigen that can induce oral tolerance and could, therefore, be a novel therapeutic modality for patients with IBD.

## Methods

### Mice

CB-17 syngeneic severe combined immunodeficiency (SCID) and BALB/c (H-2d, IA-IE) female mice, bred under specific pathogen-free conditions in accredited animal facilities, were purchased from CLEA Japan, Inc. (Tokyo, Japan). All mice used in this study were 8–12 weeks of age, fed standard laboratory chow, and maintained in the animal center at the Ehime University Graduate School of Medicine (Ehime, Japan) under controlled conditions (22 °C, 55% humidity, and 12-h day/night cycle). Animals were randomly assigned to experimental groups, and each cage contained animals of all groups.

### Antigens and feeding regimens

Animals were orally administered hCA I (0.3 mg/day) or keyhole limpet hemocyanin (KLH) (0.3 mg/day; 77600; Thermo Fisher Scientific, Rockford, IL, United States) in phosphate-buffered saline (PBS) for seven days. The total daily dose of hCA I or KLH delivered by the continuous feeding regimen was calculated based on the average consumption (5 mL/day). Bottles containing hCA I or KLH in PBS were changed twice daily to avoid contamination. The dosage of the protein was set based on existing reports and our efficacy analyses results^37^. Control groups received PBS for seven consecutive days. In the rectal administration experiment, hCA I and KLH were diluted and purified in PBS to 0.3 mg in 200 μL and gently administered rectally using a rectal administration tube (C30PU-MRE1711; Instech, PA, United States) once a day. The control group received 200 µL of PBS through the rectum once a day.

### Induction of colitis

Seven days after sensitization, colitis was induced according to previously described methods^38^, with some modifications. Briefly, CD4^+^CD25^−^ T cells were isolated from the spleens of BALB/c mice using the CD4^+^CD25^+^ Regulatory T Cell Isolation Kit and AutoMACS (Miltenyi Biotec, Bergisch Gladbach, Germany). CD4^+^CD25^−^ T cells (3 × 10^5^ cells/mouse) were suspended in 0.2 mL of PBS and intraperitoneally injected into SCID mice. Control SCID mice were injected with 0.2 mL of PBS alone. The day of this transfer was designated as Day 0.

### Preparation of CA I in feces and colon tissue and immunoprecipitation

The feces of SCID mice, used in the oral administration experiment, were collected daily to quantify fecal CA I protein content. Briefly, the feces from three animals from each group were collected daily, of which 400 mg was used for further experimentation. A 1-cm section of the large intestine, proximate to the cecum, was excised from a normal BALB/c mouse to obtain mCA I, which was used as the control. The colon tissue and feces sample were washed thrice using sterile PBS and individually placed in 10 mL PBS with 1.0 mm silica spheres (6912; Lysing Matrix C; MP Biomedicals, Solon, OH, United States). After vortexing for 5 min, the silica spheres and residual feces or colon tissue were removed by centrifugation at 5000×*g* for 5 min at 4 °C. Subsequently, 500 μL of the recovered supernatant was added to 1,000 μL of PBS and centrifuged at 18,000 × *g* for 30 min at 4 °C. The CA I protein was extracted from the lysate using the Dynabeads™ Protein G Immunoprecipitation Kit (DB10007; Thermo Fisher Scientific). Then, 2 μg of CA I polyclonal antibody (13198-2-AP; Proteintech Group Inc., Rosemont, IL, United States) diluted in 200 µL of Ab Binding and Washing Buffer was added to the magnetic beads. After binding the antibody to the beads, 250 μL of the supernatant of each sample was added. Finally, a non-denaturing elution was performed to recover only the target protein. For isotype control confirmation experiments, mouse (G3A1) mAb IgG 1 isotype control (5415; Cell Signaling, Massachusetts, USA) was used. Reproducibility was confirmed with another carbonic anhydrase 1 antibody (GTX83196; Gene Tex, CA, USA). Coomassie Brilliant Blue (CBB) staining was performed using 4–12% Bolt® Bis–Tris plus gel (Thermo Fisher) and NuPAGE™ MES SDS running buffer (Invitrogen) to confirm the uniformity of the samples after immunoprecipitation.

### Immunoblot analysis

The target protein, recovered from feces by immunoprecipitation, was separated using the Jess system (automated simple western blot system; ProteinSimple, San Jose, CA, United States), with a 12–230 kilodaltons Separation Module (#SM-W-004), and detected using CA I polyclonal antibody (13198-2-AP; Proteintech Group Inc.) and an Anti-Rabbit Detection Module (#DM-001), according to the manufacturer’s instructions. The RePlex feature and Total Protein Assay were employed to determine the total protein amount within the same capillary, which was used for normalization (RP-001 and DM-TP1; Protein Simple, Bio-Techne, CA, USA). The data analysis was performed using Compass software for Simple Western (version: 4.1.0).

### Pilot drugs used for the treatment of IBD

Prednisolone (PSL; PubChem CID: 5755) and 5-aminosalicylic acid (5-ASA; also known as mesalazine; PubChem CID: 4075) were purchased. 5-ASA (100 mg kg^–1^ day^–1^) and PSL (2 mg kg^–1^ day^–1^) were used to make comparisons between the hCA I and PBS groups. These doses were set as effective concentrations based on previous literature^39,40^. The drugs were dissolved in PBS and administered via free-choice drinking. The bottle was replaced every day to prevent contamination and drug denaturation.

### Microbiota processing and analysis

The fresh feces of SCID mice, used in assessing the efficacy of established drug treatments, were collected daily to quantify intestinal microbiota content. Control mice were given water to drink. To avoid cage effects, one mouse from each group was kept in a cage, and fecal samples were collected. Briefly, the feces from three animals in each group were collected daily and used for further experimentation. The fecal microbiota samples from each group were suspended in the Genefind 2.0 (A41499; Beckman Coulter, CA, United States) lysis buffer. The suspension was transferred to a MORA (46211; AMR Inc., Tokyo, Japan) bead tube and crushed for 3 min. After centrifugation, the supernatant was automatically purified using Genefind 2.0 (Beckman Coulter) according to the manufacturer’s protocol, and DNA was eluted with 80 µL of sterile water. The V3–V4 region of the bacterial 16S ribosomal RNA gene was amplified using the KAPA HiFi HotStart PCR kit and the barcode-indexed primers 341F (CCTACGGGNGGCWGCAG) and 806R (GACTACHVGGGTATCTAATCC). The amplicons were purified using AmpureXP (A63880; Beckman Coulter) and quantified using Qubit (Q33238; Thermo Fisher Scientific). 16S rRNA sequencing was performed using the MiSeq™ system (SY-410–1003; Illumina, San Diego, CA, United States). The 16S rRNA sequence analysis was performed using the QIIME2 suite of software tools (v3.5.3)^41^. Operational taxonomic units with 97% sequence similarity were selected and the sequences were aligned to the Silva database (v138.1)^42^. For comparison of beta diversity, weighted and unweighted UniFrac distances were calculated^43^. Attach the raw data (Supplementary File 1).

### Histological assessment of colitis

Mice were euthanized 4 weeks after cell transfer; subsequently, their ascending colons were excised, fixed with 10% neutral-buffered formalin, and embedded in paraffin. Thin tissue sections were then subjected to H&E staining or periodic acid Schiff staining. Histological assessment of colitis was scored as previously described (Supplementary Table S2)^38^.

### Ex vivo MLNs culture and measurements of MLNs cytokine levels

Mesenteric lymph node (MLN) cells (1 × 10^6^) were cultured in complete Roswell Park Memorial Institute (RPMI) 1640 Medium (containing 10% fetal bovine serum (FBS), 20 mM HEPES, 2-Mercaptoethanol (2-ME), penicillin, and streptomycin; Life Technologies Japan Ltd., Tokyo, Japan) for 72 h. Thereafter, 25 ng/mL phorbol 12-myristate 13-acetate (PMA; P88139; Sigma-Aldrich, St Louis, MO, United States) and 1 μg/mL ionomycin (10,634; Sigma-Aldrich) were added. Once culturing was completed, the supernatants were assayed for IL-6 (M6000B), IL-17 (M1700), IFN-γ (MIF00), TNF-α (MTA00B), transforming growth factor-β1 (TGF-β1) (DB100C), IL-10 (M1000B), and MCP-1 (DY479-05) using ELISA kits (R&D Systems, Inc., Minneapolis, MN, United States).

### Ex vivo colon culture and colon cytokine levels

Cytokine concentrations from colon culture were assessed as described previously^14,15^. Briefly, after feces were removed, 1-cm sections were excised from the transverse colon and washed thrice using sterile PBS. The colon tissue sections were placed into complete RPMI 1640 medium and cultured for 72 h. Thereafter, the supernatants were assayed for cytokines, namely IL-6, IL-17, IFN-γ, TNF-α, TGF-β1, and IL-10, using ELISA kits (R&D Systems). For comprehensive analysis, the supernatants (n = 3) collected from each group were mixed and examined using the Mouse Cytokine Array Panel A (ARY006; R&D Systems). The analysis was performed using Image Quant TL (Cytiva, Tokyo, Japan).

### RNA extraction from the colon and MLNs and quantitative real-time polymerase chain reaction

Transverse colon specimens and MLNs were homogenized with TissueLyser (QIAGEN, Tokyo, Japan). Total RNA was isolated using the RNeasy Plus Mini Kit (74,134; QIAGEN, Tokyo, Japan). Thereafter, cDNA was generated using the High-Capacity cDNA Reverse Transcription Kit (4,368,814; Thermo Fisher Scientific). Quantitative real-time PCR (qPCR) was employed for IL-6, IL-10, IL-17A, TGF-β, FOXP3, RORγt, and ALDH1A2 assays using mRNA from the MLNs and colons of colitic mice. Hypoxanthine phosphoribosyltransferase 1 (HPRT1) expression served as the control. qPCR was performed using a LightCycler 96 Real-Time PCR System (Roche, Basel, Switzerland) with LightCycler FastStart DNA Master SYBR Green I (Roche, Basel, Switzerland). The primer sequences used in these analyses are presented in Supplementary Table S3.

### Immunohistochemistry

All sections were deparaffinized and rehydrated. Sections for CA I detection were autoclaved at 110 °C for 1 min in 10 mM citrate buffer solution (pH 6.0). After blocking for endogenous peroxidase with 0.03% hydrogen peroxide, the sections were incubated with rabbit anti-CA I polyclonal antibody (1:500 dilution; NNP188191; Novus Biologicals, CO, United States) overnight at 4 °C. The sections were subsequently incubated at 25 °C for 40 min with goat anti-mouse immunoglobulin conjugated to a peroxidase-labeled amino acid polymer, as provided in the MAX-PO (R) Kit (424142; Nichirei Corp., Tokyo, Japan). Tissue sections were then incubated with Simple Stain DAB Solution (415172; Nichirei) and counterstained with hematoxylin.

### Flow cytometric analysis and intracellular cytokine synthesis analysis

MLN cells or spleen cells were collected (n = 3). After blocking the Fc receptors with purified rat antimouse CD16/CD32 (93; Invitrogen., Tokyo, Japan), frequencies of forkhead box protein 3 (Foxp3) + CD4 + CD25 + Tregs were determined using the Anti-Mouse/Rat Foxp3 Staining Set (77-5775-4-; eBioscience, San Diego, CA), allophycocyanin-conjugated anti-Foxp3 mAb (FJK-16s), PE-conjugated anti-CD25 mAb (PC61.5), and FITC-conjugated anti-CD4 mAb (RM4-5). The stained cells were analyzed using a flow cytometer (FACSCalibur; BD Bioscience). Data were processed using CellQuest software (BD Bioscience). Fluorescence staining was analyzed by fluorescence-activated cell sorting using FlowJo software version 10.0 (FLOWJO, LLC, Ashland, OR).

### Lipocaline-2 ELISA

The abundance of lipocalin-2 in feces was determined by ELISA using mouse lipocalin-2/NGAL detection kit (MLCN20; R&D) according to the manufacturer’s instruction.

### Statistical analysis

Data from all individual experiments are expressed as mean values ± standard deviation (SD). Where appropriate, Student’s *t*-test was used. For multiple comparisons, a one-way analysis of variance (ANOVA) followed by Tukey’s honestly significant difference test was performed. All statistical analyses were calculated using JMP software (SAS Institute Inc., Cary, NC, United States). Differences were considered statistically significant when *P* < 0.05.

### Ethical considerations

Experimental protocols were approved by the Committee of Animal Experimentation of Ehime University Graduate School of Medicine (Ehime, Japan) and performed according to laboratory practice guidelines (Institutional review board approvals 05-TI-67-1). All studies were performed in compliance with the ARRIVE guidelines.

## Supplementary Information

Supplementary Information 1.

Supplementary Figure 1.

Supplementary Figure 2.

Supplementary Figure 3.

Supplementary Figure 4.

Supplementary Figure 5.

Supplementary Figure 6.

Supplementary Information 2.

Supplementary Table 1.

Supplementary Table 2.

Supplementary Table 3.

## Data Availability

The datasets generated during and/or analyzed during the current study are available from the corresponding author upon reasonable request.

## Abbreviations

AA: Amino acid
ALDH1A2: Aldehyde dehydrogenase 1 family member A2
ANOVA: Analysis of variance
APC: Antigen-presenting cell
APC_CA I 58__–__73_: APC pulsed with carbonic anhydrase I 58–73
BIC: *Brevibacillus *in vivo Cloning
CA I: Carbonic anhydrase I
CBA: Cecal bacterial antigen
CBB: Coomassie brilliant blue
CD: Crohn's disease
cDNA: Complementary DNA
CXCL9: C-X-C motif chemokine ligand 9
CXCL10: C-X-C motif chemokine ligand 10
CXCR3: C-X-C motif chemokine receptor 3
DCregs: Regulatory dendritic cells
DCs: Dendritic cells
DSS: Dextran sulfate sodium
ELISA: Enzyme-linked immunosorbent assay
FBS: Fetal bovine serum
FITC: Fluorescein isothiocyanate
Foxp3: Forkhead box protein 3
GM-CSF: Granulocyte macrophage-colony stimulating factor
hCA I: Human carbonic anhydrase I
HPRT1: Hypoxanthine phosphoribosyltransferase 1
IEC: Intestinal epithelial cell
IFN-γ: Interferon-gamma
IL: Interleukin
KLH: Keyhole limpet hemocyanin
LPS: Lipopolysaccharide
MHC: Major histocompatibility complex
MLN: Mesenteric lymph node
mRNA: Messenger RNA
NEMO^tamIEC-KO^: Tamoxifen-inducible IEC-specific inhibition of NF-κB through conditional ablation of IKK subunits
PAS: Periodic acid Schiff
PBS: Phosphate-buffered saline
PCR: Polymerase chain reaction
PE: Phycoerythrin
PerCP: Peridinin chlorophyll protein
PMA: Phorbol 12-myristate 13-acetate
PSL: Prednisolone
pTregs: Peripheral regulatory T cells
qPCR: Quantitative real-time PCR
RORγt: Retinoic acid-related orphan receptor gamma t
RPMI: Roswell Park Memorial Institute
SCID: Severe combined immunodeficiency
SD: Standard deviation
SDS-PAGE: Sodium dodecyl sulfate–polyacrylamide gel electrophoresis
SPF: Specific pathogen-free
TGF-β: Transforming growth factor-β
Th0: Antigen-specific CD4^+^ helper T cells
Th1: CD4^+^ T cells producing IFN-γ
Th17: CD4^+^ T cells producing interleukin-17
TNF: Tumor necrosis factor
Tregs: Regulatory T cells
UC: Ulcerative colitis
2-ME: 2-Mercaptoethanol
5-ASA: 5-Aminosalicylic acid
