# Supplementary figures and images for: Oral administration of human carbonic anhydrase I suppresses colitis in a murine inflammatory bowel disease model
Source: Sci Rep. 2022 Oct 26;12:17983. doi: 10.1038/s41598-022-22455-y (PMC9606376; doi:10.1038/s41598-022-22455-y)

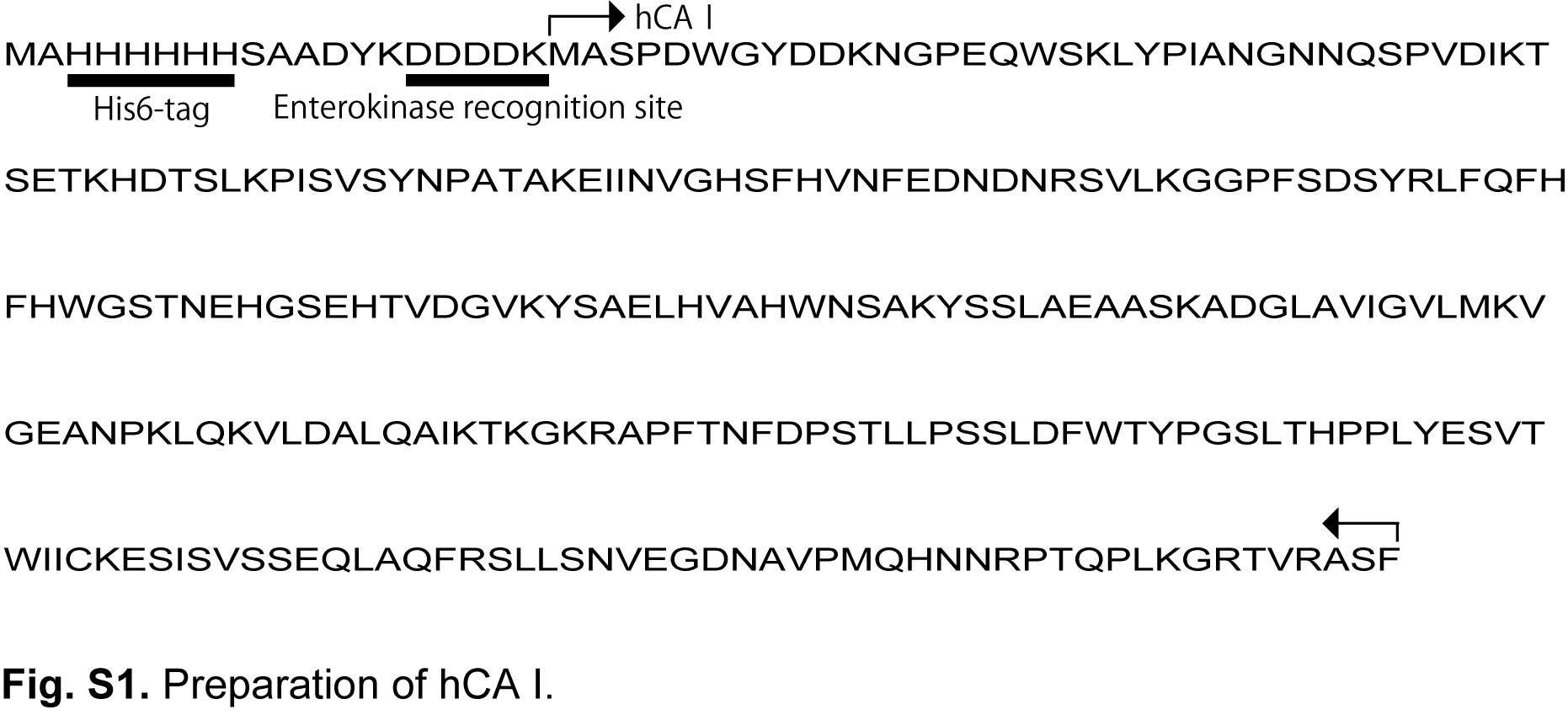

Supplement: Supplementary file 2 — Supplementary Figure 1. [file 41598_2022_22455_MOESM2_ESM.tif]

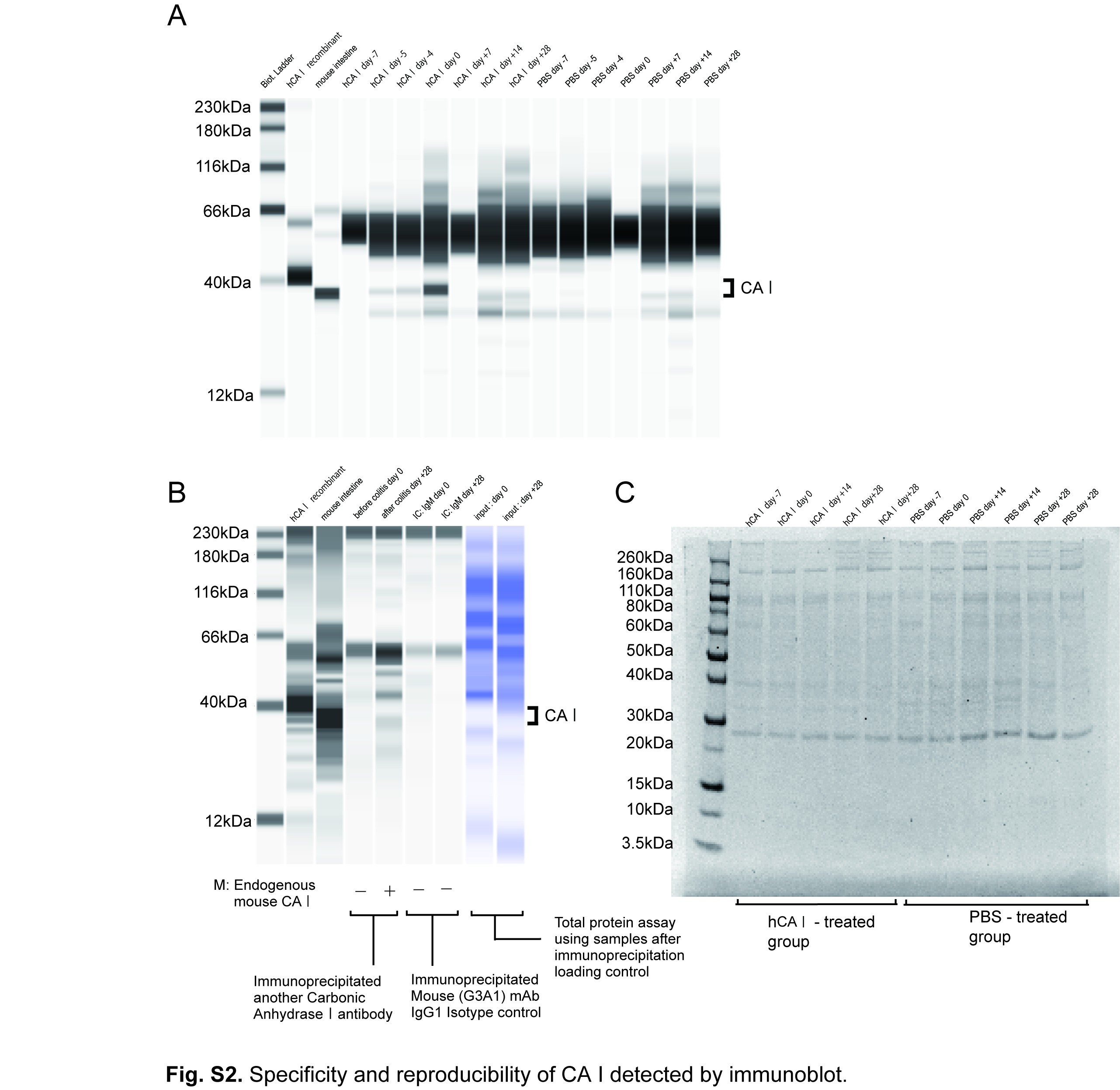

Supplement: Supplementary file 3 — Supplementary Figure 2. [file 41598_2022_22455_MOESM3_ESM.tif]

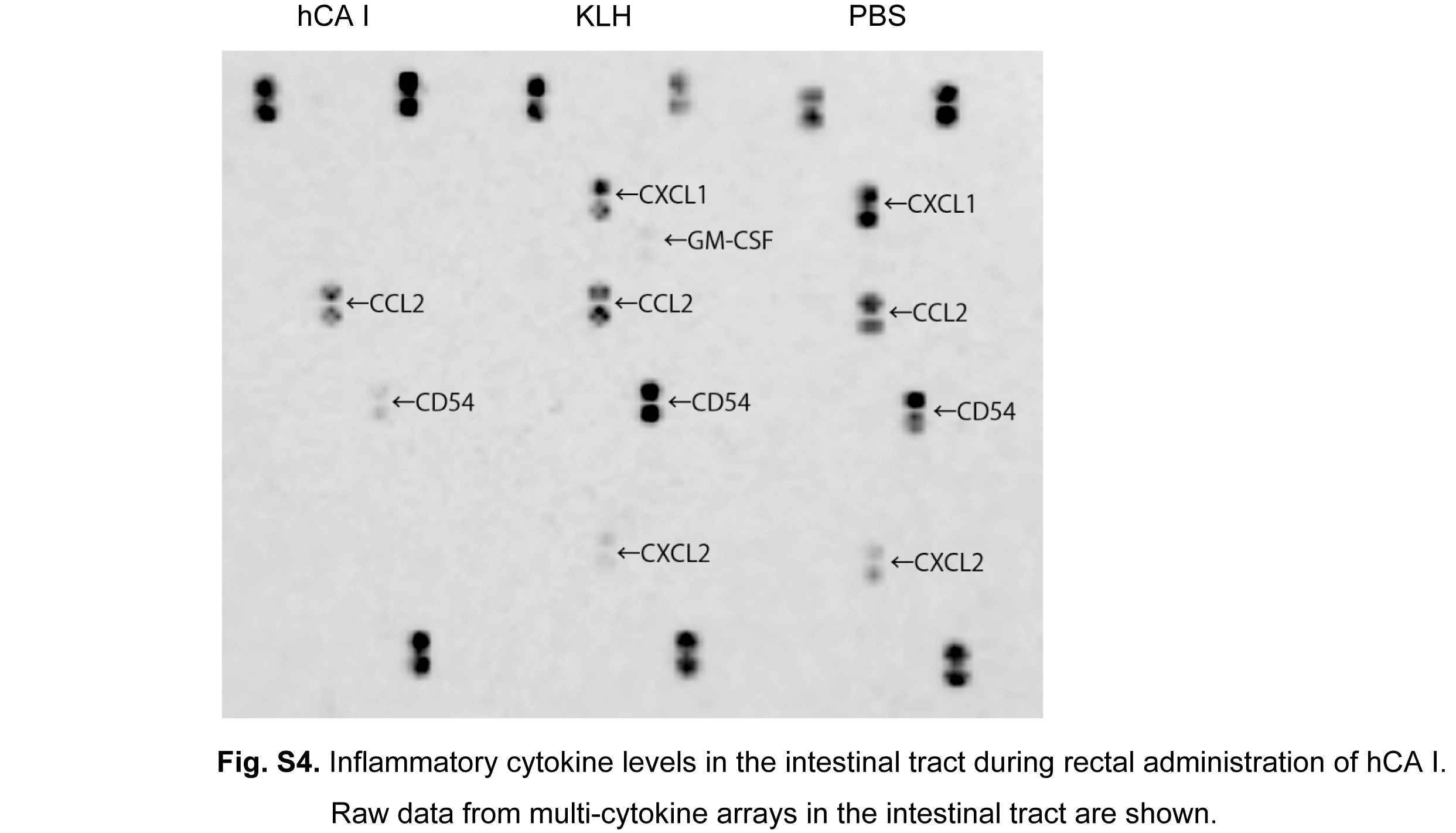

Supplement: Supplementary file 5 — Supplementary Figure 4. [file 41598_2022_22455_MOESM5_ESM.tif]

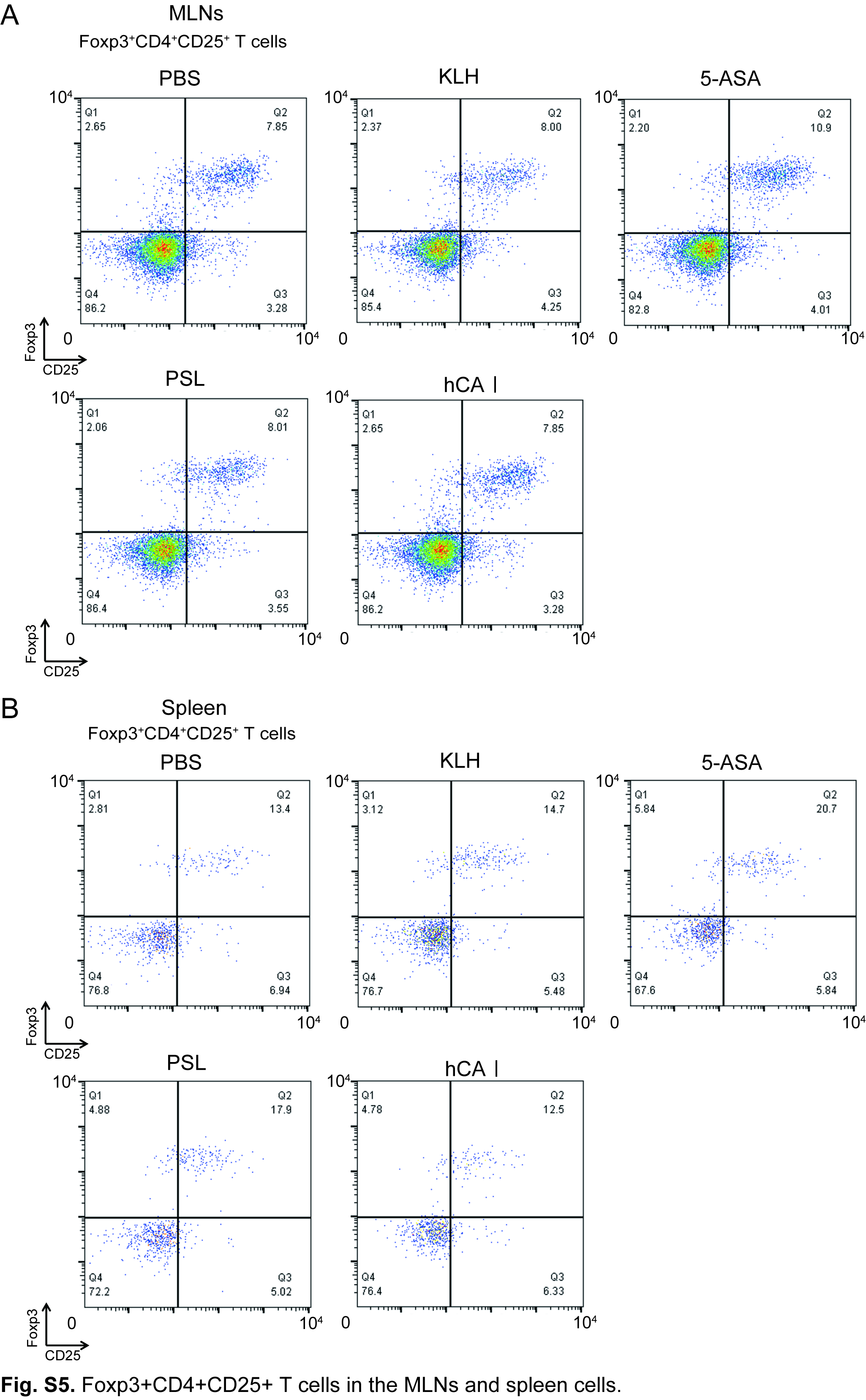

Supplement: Supplementary file 6 — Supplementary Figure 5. [file 41598_2022_22455_MOESM6_ESM.tif]

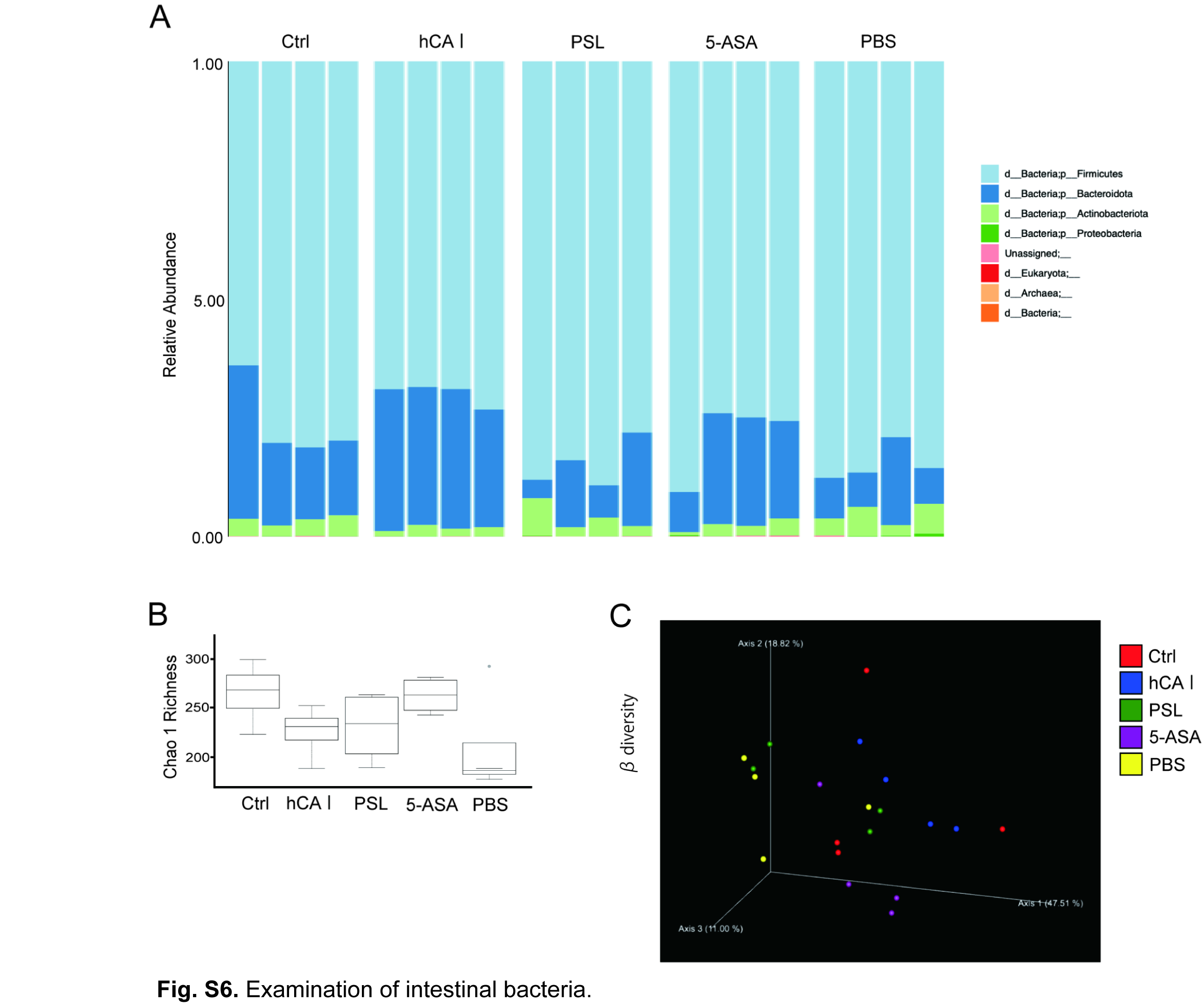

Supplement: Supplementary file 7 — Supplementary Figure 6. [file 41598_2022_22455_MOESM7_ESM.tif]
