# Supplementary material for: Oral administration of human carbonic anhydrase I suppresses colitis in a murine inflammatory bowel disease model
Source: Sci Rep. 2022 Oct 26;12:17983. doi: 10.1038/s41598-022-22455-y (PMC9606376; doi:10.1038/s41598-022-22455-y)
Supplement: Supplementary file 4 — Supplementary Figure 3. [file 41598_2022_22455_MOESM4_ESM.docx]

**Supplementary Methods:**

***Preparation of Human Carbonic Anhydrase I***

hCA I complementary DNA (cDNA) was amplified using polymerase chain reaction (PCR). Amplified DNA was inserted into pBICs plasmids (modified pBIC1; Higeta Shoyu Co., Ltd., Tokyo, Japan) using the *Brevibacillus in vivo* Cloning (BIC) method of the *Brevibacillus* Expression system (HB116; Takara Bio Inc., Shiga, Japan) to generate the construct pBIC-hCA I, which expressed N-terminal MA, His6 tag, SAADYK, and the enterokinase recognition site (DDDDK). The final amino acid sequence of hCA I is presented (see Supplementary Fig. S1, which describes the preparation of hCA I). Briefly, the expression vector pBICs-hCA I was used to transform the *Brevibacillus* SP3 strain. The resultant transformants were pre-cultured in 2SYN medium (for 1 d at 30°C), and then the main culturing (1% inoculation from pre-culture in 3 × 100-mL flasks for 2 d at 30°C) was performed. After culturing was completed, the pellet obtained, following the collection of the 300 mL culture solution, was washed thrice with 300 mL buffer A (20 mM sodium phosphate; 500 mM sodium chloride; pH 7.4), and another 300 mL buffer A was added before sonication. Thereafter, the lysate was centrifuged at 12,000 × *g* for 40 min to collect the soluble fraction, which was sterilized with a 0.22-µm filter. The recovered soluble fraction was purified using HisTrap HP (2 × 5 mL; 17524802; GE Healthcare Bio-sciences Corp., Piscataway, NJ, United States). The bound protein was eluted from the HisTrap HP column with imidazole (0 mM → 500 mM; 200 mL) using the AKTA chromatography system (AKTA explorer 10s; GE Healthcare Bio-Sciences Corp.). The eluted fraction was collected and dialyzed against a designated buffer (20 mM sodium phosphate; pH 7.4). Sodium dodecyl sulfate-polyacrylamide gel electrophoresis (SDS-PAGE) was performed, and the target protein was detected by Coomassie brilliant blue (CBB) staining and western blot using Real Gel Plates. As a result, target protein purity was confirmed, and the fractions were collected. The prepared hCA I protein was purchased from Higeta Shoyu Co., Ltd. (Tokyo, Japan) or ProteinExpress Co., Ltd. (Chiba, Japan).

**Supplementary Data**

**Supplementary Figure 1. *Preparation of h******CA I.*** hCA I amino acid sequence. The His6 tag was removed by the enterokinase recognition sequence.

**Supplementary Figure 2. *Specificity and reproducibility of CA I detected by immunoblot.*** (A) Raw data are shown (Figure 3). (B) The detectability of CA I by isotype control was analyzed using immunoblot, and the band was no longer detected by immunoblot with IgG, indicating that the band is specific for CA I. To confirm reproducibility, experiments were performed with other CA I antibodies for immunoprecipitation (IP). As a loading control, the results of the total protein detection assay with immunoblot after IP are also shown. (C) CBB staining for loading control of sample after IP are shown.

**Supplementary Figure 3. *Rectal administration of hCA I before induction of colitis failed to inhibit the induction of colitis.*** (A) Macroscopic findings of the colon after administration of the Indigocarmine-stained drugs through the rectum. (B) Experimental protocol (see *Methods*). (C) Histological scores on day 28. Horizontal bars: median. CD4^+^CD25^-^ T cell (3 × 10^5^) transfer model mice (n = 5 mice/model) were treated with PBS, KLH, and hCA I indicated as triangles, squares, and black circles, respectively. Data shown are from single experiments.

**Supplementary Figure 4. *Inflammatory cytokine levels in the intestinal tract during rectal administration of hCA I.*** Raw data from multi-cytokine arrays in the intestinal tract are shown.

**Supplementary Figure 5. *Foxp3^+^CD4^+^CD25^+^ T cells in the MLNs and spleen cells.*** (A) The frequency of Foxp3^+^CD4^+^CD25^+^ T cells in the MLNs from CD4^+^CD25^-^ T-cell-transferred mice treated with hCA I, KLH, PSL, 5-ASA, and PBS was analyzed by flow cytometry; The expression of Foxp3^+^CD4^+^CD25^+^ T cells in the MLN cells are represented as dot plots. (B) The frequency of Foxp3^+^CD4^+^CD25^+^ T cells in the spleen is shown. Tissues from a total of three mice per group were mixed and dissected on day 28. Data shown are representative of two independent experiments.

**Supplementary Figure 6. *Examination of intestinal bacteria.*** (A) Experimental protocol (see *Methods*). Bacterial composition at phylum level. The relative amount of bacterial composition at the phylum level in stool from colon is shown on day 0 (n=4). (B) Alpha diversity in stool of each group measured by Chao1 richness index. (C) Beta diversity in stool of each group measured by principal coordinate analysis (PCA) of weighted UniFrac analysis.
