# Supplementary material for: Oral administration of human carbonic anhydrase I suppresses colitis in a murine inflammatory bowel disease model
Source: Sci Rep. 2022 Oct 26;12:17983. doi: 10.1038/s41598-022-22455-y (PMC9606376; doi:10.1038/s41598-022-22455-y)
Supplement: Supplementary file 9 — Supplementary Table 1. [file 41598_2022_22455_MOESM9_ESM.docx]

Supplementary Table 1.

The difference in amino acid sequence between mouse and human CA I

| Mouse CA I | 1 MASADWGYGSENGPDQWSKLYPIANGNNQSPIDIKTSEANHDSSLKPLSISYNPATAKEI 60 |
| --- | --- |
| Human CA I | 1 MASPDWGYDDKNGPEQWSKLYPIANGNNQSPVDIKTSETKHDTSLKPISVSYNPATAKEI 60 |
|  |  |
| Mouse CA I | 61 VNVGHSFHVIFDDSSNQSVLKGGPLADSYRLTQFHFHWGNSNDHGSEHTVDGTRYSGELH 120 |
| Human CA I | 61 INVGHSFHVNFEDNDNRSVLKGGPFSDSYRLFQFHFHWGSTNEHGSEHTVDGVKYSAELH 120 |
|  |  |
| Mouse CA I | 121 LVHWNSAKYSSASEAISKADGLAILGVLMKVGPANPSLQKVLDALNSVKTKGKRAPFTNF 180 |
| Human CA I | 121 VAHWNSAKYSSLAEAASKADGLAVIGVLMKVGEANPKLQKVLDALQAIKTKGKRAPFTNF 180 |
|  |  |
| Mouse CA I | 181 DPSSLLPSSLDYWTYFGSLTHPPLHESVTWVICKDSISLSPEQLAQLRGLLSSAEGEPAV 240 |
| Human CA I | 181 DPSTLLPSSLDFWTYPGSLTHPPLYESVTWIICKESISVSSEQLAQFRSLLSNVEGDNAV 240 |
|  |  |
| Mouse CA I | 241 PVLSNHRPPQPLKGRTVRASF 261 |
| Human CA I | 241 PMQHNNRPTQPLKGRTVRASF 261 Homology＝78.2％ |
