# Supplementary material for: Oral administration of human carbonic anhydrase I suppresses colitis in a murine inflammatory bowel disease model
Source: Sci Rep. 2022 Oct 26;12:17983. doi: 10.1038/s41598-022-22455-y (PMC9606376; doi:10.1038/s41598-022-22455-y)
Supplement: Supplementary file 10 — Supplementary Table 2. [file 41598_2022_22455_MOESM10_ESM.doc]

Supplementary Table 2.

Histological Scoring System for Colonic Section in CD4^+^CD25^−^ T cell transfer Colitis Model

| Parameter | Score | Description |
| --- | --- | --- |
| Severity of inflammation | 0 | None |
|  | 1 | Mild lymphoid infiltration |
|  | 2 | Marked lymphoid infiltration. Focal degeneration of crypts |
|  | 3 | Severe inflammation, multifocal crypt degeneration and/or erosions |
| Extent of inflammation | 0 | None |
|  | 1 | Mucosal |
|  | 2 | Submucosal |
| Amount of mucus | 0 | Normal |
|  | 1 | Slight decrease of mucus |
|  | 2 | Moderate decrease. Focal absence of mucus |
|  | 3 | Severe depletion of mucus. |
|  | 4 | Total absence of mucus |
| Degree of proliferation | 0 | None |
|  | 1 | Mild increase in cell numbers and crypt length |
|  | 2 | Moderate increase. Focally marked increase. |
|  | 3 | Marked increase - entire section |

The sum of the four individual parameters constitutes the total scoring range of 0–13.
