# Supplementary material for: Oral administration of human carbonic anhydrase I suppresses colitis in a murine inflammatory bowel disease model
Source: Sci Rep. 2022 Oct 26;12:17983. doi: 10.1038/s41598-022-22455-y (PMC9606376; doi:10.1038/s41598-022-22455-y)
Supplement: Supplementary file 11 — Supplementary Table 3. [file 41598_2022_22455_MOESM11_ESM.doc]

Supplementary Table. 3

| Sequences of the primers used in RT-PCR | | |
| --- | --- | --- |
| HPRT1 | forward | 5’- TCCTCCTCAGACCGCTTTT -3’ |
|  | reverse | 5’- CCTGGTTCATCATCGCTAATC -3’ |
| IL-10 | forward | 5′- GGTTGCCAAGCCTTATCGGA -3′ |
|  | reverse | 5′- ACCTGCTCCACTGCCTTGCT -3′ |
| IL-6 | forward | 5′- ACAACCACGGCCTTCCCTACTT -3′ |
|  | reverse | 5′- CACGATTTCCCAGAGAACATGTG -3′ |
| IL-17A | forward | 5′- TGTGAAGGTCAACCTCAAAGTC -3′ |
|  | reverse | 5′- AGGGATATCTATCAGGGTCTTCATT -3′ |
| TGF-β | forward | 5′- TGACGTCACTGGAGTTGTACGG -3′ |
|  | reverse | 5′- GGTTCATGTCATGGATGGTGC -3′ |
| Foxp3 | forward | 5′- AGAAGCTGGGAGCTATGCAG -3′ |
|  | reverse | 5′- GCTACGATGCAGCAAGAGC- 3′ |
| RORγT | forward | 5′- GCAGAACTGCCCCATTGA -3′ |
|  | reverse | 5′- GACATTCGGCCAAACTTGA -3′ |
| ALDH1A2 | forward | 5′- TGCATTCACAGGCTCTACTGA -3′ |
|  | reverse | 5′- CGAGTTCCAGGGTCACTCTC -3′ |
